# Histone 3 Lysine 27 Trimethylation Signature in Breast Cancer

**DOI:** 10.3390/ijms222312853

**Published:** 2021-11-27

**Authors:** Lidia Borkiewicz

**Affiliations:** Department of Biochemistry and Molecular Biology, Medical University of Lublin, 20-059 Lublin, Poland; lidiaborkiewicz@umlub.pl; Tel.: +48-81-4486350

**Keywords:** histone post-translational modifications, histone methylation, transcriptional regulation, gene silencing, chromatin modification

## Abstract

Cancer development and progression rely on complicated genetic and also epigenetic changes which regulate gene expression without altering the DNA sequence. Epigenetic mechanisms such as DNA methylation, histone modifications, and regulation by lncRNAs alter protein expression by either promoting gene transcription or repressing it. The presence of so-called chromatin modification marks at various gene promoters and gene bodies is associated with normal cell development but also with tumorigenesis and progression of different types of cancer, including the most frequently diagnosed breast cancer. This review is focused on the significance of one of the abundant post-translational modifications of histone 3- trimethylation of lysine 27 (H3K27me3), which was shown to participate in tumour suppressor genes’ silencing. Unlike other reviews in the field, here the overview of existing evidence linking H3K27me3 status with breast cancer biology and the tumour outcome is presented especially in the context of diverse breast cancer subtypes. Moreover, the potential of agents that target H3K27me3 for the treatment of this complex disease as well as H3K27 methylation in cross-talk with other chromatin modifications and lncRNAs are discussed.

## 1. Introduction

Cancer has become the most common cause of death across the globe, taking the life of 10 million people in 2020 [1]. Among other types, breast cancer (BC) comes top of the most frequently diagnosed (11.7% of all cancer cases), reaching 6% of mortality rate, as reported by the World Health Organization (WHO). The biology of BC shows a high rate of inter- and intratumour variability, as well as the morphology and cancer stage at diagnosis, making the effective cancer prognosis and treatment challenging [2].

Taking into account the genetic context, important risk factors for BC include the expression of BC susceptibility genes such as BRCA1 (BC gene 1) and BRCA2, yet no more than 25% of all BC cases can be linked to hereditary characteristics [3,4]. Other individual factors for instance sex, age, reproduction status, breastfeeding, and menopause, are tightly connected with hormonal background [5]. Hormone receptors (HR), especially estrogen (ER) and progesterone (PR) receptors play a crucial role in the development and progression of BC [6]. Depending on HR and other proteins such as the human epidermal growth factor receptor 2- HER2, which are expressed (or not) in cancer cells, and the number of cells expressing the proliferation marker- Ki67, five molecular BC subtypes have been described: luminal A or HR+/HER2−/Ki67 low (HR-positive/HER2-negative/Ki67 < 20%), luminal B or HR+/HER2+/−Ki67 high (HR-positive/HER2-positive/negative/Ki67 ≥ 20%), triple-negative (TNBC) or HR−/HER2− (HR/HER2-negative), HER2-overexpressed, and so-called normal-like breast cancer which closely resembles luminal A subtype [7]. The BC subtypes differ with histological and molecular characteristics, growth rate, and response to chemotherapy and endocrine therapy. Therefore, the type of treatment is administered respectively to the expression of ER, PR, and HER2 as well as tumour morphology, its grade and size, and the appearance of metastases to the lymph nodes or distant metastases [6]. In general, the presence of ER is considered a good prognostic marker used to identify tumours to treat with endocrine therapy targeting ER signalling pathways. HER2+ subtype tumours can be treated by anti-HER2 monoclonal antibodies targeting HER2-dependent signalling. The TNBC subtype exhibits the worst prognosis subtype, as targeted therapeutic options are not available, thereby surgery, cytotoxic drugs, or radiation therapy are the remaining options of therapy [8,9].

At the molecular level, BC is driven by taking control over various transduction pathways responsible for cell cycle, metabolism, differentiation, and apoptosis [10]. For example, Wnt, Notch, Hedgehog, and several other signalling pathways involved in the epithelial-mesenchymal transition (EMT) are hijacked by tumour cells for metastasis formation [11]. During EMT, epithelial cells form independent motile mesenchymal-like cells that can disseminate to distal organs. Furthermore, at the metastatic site, cells are stimulated to undergo the reversal mesenchymal-epithelial transition (MET) that reinstates proliferation and allows collective outgrowth [12]. The often observed phenomena in cancer cells-alterations in the expression of proteins that are involved in cell signalling are caused by deregulation of epigenetic mechanisms like DNA methylation, histone modifications, and the activity of long non-coding RNAs (lncRNAs, ncRNAs made of more than 200 nucleotides), which directly or indirectly promote or repress the gene transcription [10].

In gene regulation and other chromatin-related processes, histone post-translational modifications (PTMs) play a fundamental role, as the core histones H2A, H2B, H3, and H4 wrapped by 147 bp of DNA form the basic unit of chromatin [13]. The exposed N-terminal tales as well as their structural globular domains undergo various modifications such as acetylation (ac) of lysines (K) and methylation (me) of K and arginines (R), phosphorylation, ubiquitylation, glycosylation, sumoylation, ADP-ribosylation, and carbonylation [14]. Most post-translational histone modifications were identified in H3 and H4 and are considered as chromatin marks that influence genome organization and gene activity in the presence of other proteins including transcription factors [15,16]. Not surprisingly, aberrations within histone PTMs are found in various cancers [14]. This review briefly summarizes existing data on the importance of one of the most abundant post-translational modification in cancer, H3- trimethylation of lysine 27 (H3K27me3) in BC development, metastasis, and patient outcome. In particular, the status of this histone mark in BC subtypes, its influence on the transcriptional regulation of the specific genes and the H3K27me writers and erasers are highlighted. Finally, the classes of agents that inhibit H3K27 methylation and their potential for BC treatment, as well as the interaction of H3K27me3 with other chromatin modifications and lncRNAs, have been discussed to better understand the role of epigenetics in breast carcinogenesis.

## 2. Gene Transcriptional Regulation via H3K27 Methylation

Acetylation and methylation are the most frequently studied histone PTMs [15]. Methylation of H3 occurs in its N-terminal tail at four lysine residues: K4, K9, K27, and K36 which are primary targets of specific histone methyltransferases introducing methyl group(s) into the protein (also called as histone methylation ‘writers’) and demethylases which remove the modification (‘erasers’) [15]. Other identified H3 methylated residues are K37, K79, and K122, as well as several arginines located in proximity to the methylated lysines. The H3 N-terminal K27 can be monomethylated (me1), dimethylated (me2), or trimethylated (me3) depending on the cellular context. None of the mentioned above states changes the charge of the amino acid side chain, as refers to histone acetylation, which by removing the positive charge exposes negatively charged DNA [15], therefore the function of H3K27me seems to depend on the effect on molecules that specifically recognize the methylated site called ‘readers’ [17]. Recognition of methylated residues occurs via methyl-lysine binding motifs, such as PHD (plant homeodomain), chromo, tudor, WD40, ankyrin repeat domains present in ‘readers’ proteins, which are also capable of distinguishing the methylation state of the target methyllysines and the surrounding amino acid sequence [17].

**Figure 1:**
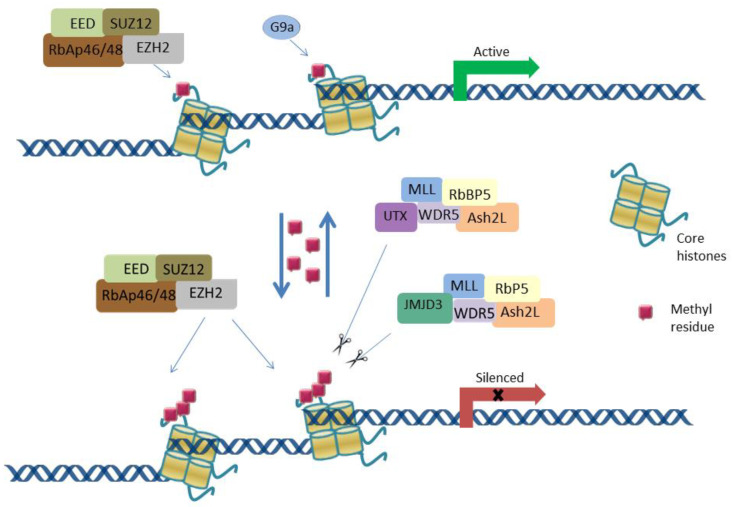
Gene transcriptional regulation via H3K27 methylation. Transcriptional repression regulated by the polycomb repressive complex 2 (PRC2) including EED, SUZ12, RbAP46/48, and EZH2, which catalyzes H3K27 mono-, di- and tri-methylation. Enrichment of H3K27 trimethylated marks in gene promoters leads to a more condensed chromatin state and transcriptional silencing of tumour suppressor genes, inflammation- and immunological response genes, and epithelial-mesenchymal transition- suppressor genes enabling tumour progression and metastasis. G9a catalyzes monomethylation of H3K27. UTX and JMJD3 demethylases form complexes with MLL, RbBP5, and WDR5, and remove methyl groups from H3K27me3, what leads to transcriptionally active chromatin. Core histones presented as golden rollers, methyl groups presented as red squares. EED (embryonic ectoderm development), SUZ12 (suppressor of zeste 12), RbAP46/48 (retinoblastoma suppressor associated protein 46/48), EZH2 (enhancer of zeste homolog 2), G9a (euchromatic histone lysine methyltransferase 2), UTX (ubiquitously transcribed X-chromosome tetratricopeptide repeat protein), JMJD3 (jumonji domain containing-3), MLL (mixed-lineage leukemia), RbBP5 (RB-binding protein 5), WDR5 (WD repeat-containing protein).

The methyltransferases targeting H3K27 are the enhancer of zeste homolog 2 (EZH2) and G9a or euchromatic histone lysine methyltransferase 2 (EHMT2), while the jumonji domain containing-3 (Jmjd3, KDM6B) and ubiquitously transcribed X-chromosome tetratricopeptide repeat protein (UTX, KDM6A) have been identified as H3K27 demethylases [18,19,20,21] (Figure 1).

Protein lysine methyltransferases (KMTs) use S-adenosyl-L-methionine (SAM) as methyl donor and transfer one to three methyl groups to the target residue of protein substrates [22]. The best known H3K27 KMT- EZH2 catalyzes its mono-, di- and trimethylation [23]. EZH2 is a member of the polycomb group (PcG) of proteins that regulate cellular differentiation and stem cell identity. Most PcG proteins are part of two transcriptional repressive complexes, termed PRC1 and 2 [24]. EZH2 or EZH1 together with an embryonic ectoderm development (EED), suppressor of zeste 12 (SUZ12), and retinoblastoma suppressor associated protein 46/48 (RbAp46/48) are components of PRC2. PRC2-mediated methylation of H3K27 leads to recruitment of PRC1 and transcriptional repression due to the chromatin compaction, preventing RNA polymerase II activity or physically blocking promoter accessibility to transcription factors or chromatin remodelling complexes [25]. Mechanistically, H3K27me3 can act as a docking site for the chromobox domain (CBX) protein subunits of PRC1, thus recruiting PRC2 and PRC1 to target genes.

Additionally, G9a methyltransferase, a well-studied H3K9 methyltransferase, was also reported to methylate H3K27, in vitro and in vivo [21,26] as well as other non-histone substrates, including itself (in vitro) [27]. The G9a -/- knockout ES (embryonic stem) cells exhibited reduced H3K27me1 levels than wild-type cells, while the levels of me2 and me3 were not affected, suggesting that G9a is partially responsible for introducing a monomethyl group in H3K27 [21].

On the other hand, UTX and JMJD3 are the main H3K27 demethylases. UTX has the ability to demethylate H3K27me1/2/3, and JMJD3 to demethylate H3K27me2/3 [21]. In addition, UTX and JMJD3 form complexes with MLL (mixed-lineage leukemia, KMT2A), RbBP5 (RB-binding protein 5), Ash2L (Set1/Ash2 histone methyltransferase complex subunit), and WDR5 (WD repeat-containing protein) [28,29]. Also, minimal yet detectable lysine demethylase activity was shown for UTX paralog UTY [30]. In addition, KIAA1718 (KDM7) having demethylase activity against multiple lysine residues, was shown to demethylate H3K9 and K27 [31]. Demethylation of H3K27 leads to transcriptionally active chromatin (Figure 1).

While H3K27 methylation was shown to play a role in developmental processes [32], dysregulation of H3K27 targeting methyltransferases and demethylases leading to abnormal methylation levels of H3K27 was reported in various diseases including autoimmune diseases, rheumatoid arthritis, and cancers [33,34,35,36].

## 3. H3K27 Methylation Effects in BC

### 3.1. H3K27 Methylation Status in BC Subtypes

In the past decades, the H3K27 methylation status and its correlation with BC has been intensively studied in various models in vitro and in patients’ samples. In a representative group of BC cell lines including MCF7 and T47D (ER+ hormone sensitive cells correlating with relatively less aggressive and more benign BC characteristics), MDA-MB-231 and MDA-MB-453 (highly invasive and metastasizing ER- cells), compared to normal epithelial breast cells (MCF10A), a histone modification signatures were studied by mass spectrometry (MS) [37]. Although H3K9me2/K14ac, H3K27me2/K36me2, and H4K20me2 were predominant modifications in MCF10A cell lines, quantitative analysis of changes in methylation profiles has shown increased levels of K27me2 and K27me3 in all tested breast cancer cell lines. Likewise, the broad H3K27me3 marking across the gene body observed in MDA-MB-231 cells suggests that repression by H3K27me3 plays a role in establishing the MDA-MB-231 aggressive phenotype, as it has been reported for multiple myeloma [38]. However, the association between elevated H3K27me3 and luminal A-like tumours has been previously reported in several immunohistochemical (IHC) studies, while in basal-like, TNBC, luminal B, and ER-positive tumours with high proliferation, particularly low expression of H3K27me3 was observed [39,40]. Interestingly, it was correlated with high expression of EZH2 in basal-like and TNBC, suggesting that elevated EZH2 activity might be required for non- H3K27 methylation-related functions, such as regulation of specific transcription factors or ubiquitination, and protein degradation leading to tumour development and progression [41] (Table 1).

The more complex data were obtained by pathology tissue-quantitative MS (PAT-H-MS) in formalin-fixed paraffin-embedded patients’ samples revealed the different histone modification set up depending on the BC subtype [42]. For instance, in the luminal A-like samples, the elevated levels of histone H3 K27me3, K27me3/K36me1, and K27me2/K36me1 and low levels of K9me3, K9me3/K14ac, K36me1, K36me2, and K27me1 were observed. The opposite trends for these modifications as well as higher levels of K27me1/K36me1 and K27me2/K36me2 compared to the other clusters, with intermediate levels of K27me3, were shown for the cluster containing four out of five TNBC and three HER+ samples. Additional clusters comprising four luminal B-like samples, two HER+ samples, and one TNBC showed increased levels of K27me2 and me3, but in one cluster the modifications were accompanied by elevated levels of peptides containing acetylation either on K18 or K23 (K18(23)ac) and K27me1/K36me3, therefore using the PAT-H-MS to distinguish the profiles of H3 post-translational modifications between the luminal B, HER+, and TNBC is not straightforward [42].

### 3.2. H3K27me3 Gene Targets in BC

According to the WHO, the average 5-year survival rate for women with BC ranges from more than 90% in high-income countries to 40% in South Africa. Importantly, loss of histone methylation marks such as H3K4me2, H3K27me3, H4R3me2, and H4R3me3 was linked with a poor prognosis for BC [43,44,45]. In the case of low H3K27me3 expression, it has been associated with large tumor size, absence of ER, and presence of metastases in lymph nodes, yet there was no association with HER2 status [43]. To understand how exactly this epigenetic mark affects carcinogenesis, the gene targets of H3K27 methylation marks were investigated. In patients’ samples of the less aggressive BC subtypes (luminal A, B HER2-), a significant increase in the H3K27me3 mark was detected at promoters of genes encoding hormone receptors ERS1, −2 (estrogen receptor 1, 2) and PGR (progesterone receptor) compared with luminal B HER2+, HER2+, and basal-like subtypes, in which a decreased H3K27me3 (and an increased level of acetylated marks) was found at the promoters of the genes encoding histones’ modifying enzymes EZH2, P300 (histone acetyltransferase) and SRC3 (steroid receptor coactivator 3), and tumour suppressor BRCA1 [46]. Moreover, the decreased expression of BRCA1 caused genome-wide EZH2 re-targeting and elevated H3K27me3 levels at PRC2 target loci in both mouse embryonic stem (ES) and human MCF7 BC cells. BRCA1 deficiency inhibited ES cell differentiation and enhanced BC cell migration and invasion in an EZH2-dependent manner [46]. The cancer-related preferences of EZH2 for specific gene targets were also reflected in studies comparing the status of H3K27me3 in BC (MCF7 and MDA-MB-231) and normal human mammary epithelial cells (HMEC). In cancer cells, genes with a gain in H3K27me3 tended to have gene functions related to cell communication and cell–cell signalling, while genes with a loss of H3K27me3 tended to have functions related to development [47]. Similar phenomena were observed in prostate (Du145 and PC3) and colon cancer cell lines (HCT116 and RKO) [47].

Some studies have demonstrated that interfering with transcription factor’s (TF) histone lysine methylation can inhibit tumour progression and invasion. Among the TFs involved in proliferation which are marked by H3K27 methylation, is RUNX3, a member of the runt-related family associated with normal development and neoplasia [48]. Additionally, inactivation of RUNX3 expression has been reported in various cancers, such as prostate, lung, pancreas, and breast [48,49,50]. In gastric cancer, RUNX3 participates in TGF-β1-dependent cell growth suppression by binding to promoter p21 and inducing expression of CDKN1A (cyclin-dependent kinase inhibitor) that leads to inhibition of cancer cell proliferation [50]. RUNX3 was frequently down-regulated in BC cell lines and primary tumours of Chinese women [49]. Nine out of nineteen BC cell lines studied showed hypermethylation in the *RUNX3* promoter region and significantly lower levels of both RUNX3 mRNA and protein. Similarly, hypermethylation of the RUNX3 gene was observed in half (23 out of 44) of the primary Singaporean BC samples tested. As a consequence, low levels of RUNX3 protein were present in the majority of the samples, and in a few cases, RUNX3 was undetectable, also in matched adjacent normal breast epithelium. Remarkably, in each case, the protein was mislocalized to the cytoplasm, which was crucial for its inactivation. In in vitro and in vivo assays, RUNX3 behaved as a growth suppressor in BC cells [49]. EZH2 knockout and rescue studies have shown that both H3K27 trimethylation and histone deacetylation by HDAC1 (histone deacetylase 1) are required to down-regulate RUNX3 expression [48]. As the use of the DNA methyltransferase (DNMT) inhibitor (5-aza-2′-deoxycytidine) also promoted RUNX3 expression in several cancer cell lines, the role of DNA methylation in RUNX3 regulation cannot be omitted [48].

Similarly, the EZH2-H3K27me3 in synergy with histone deacetylation was reported to repress expression of CDKN1C in SK-BR-3 and BT-474 BC cell lines that overexpresses HER2 (and ER+) [51].

Furthermore, two members of the Forkhead box (FOX) TF family, FOXC1 and FOXO3, playing a role in differentiation and in cell cycle progression, apoptosis, and angiogenesis (respectively) were shown to be down-regulated by EZH2 via H3K27 methylation [52,53]. Transcriptional repression of *FOXC1* was an effect of the accumulation of H3K27me3 marks in the gene promoter region and diminished acetylation of H3/H4 in MDA-MB-231 cell lines. Elevated FOXC1 concentrations lead to reduced migration and invasion of BC cells in vitro and in vivo [52]. Similarly, it was shown that BRCA1, EZH2, the DNA methyltransferases DNMT1/3a/b, and H3K27me3 are recruited to the endogenous *FOXO3* promoter, modulating *FOXO3* methylation and expression in BC cells. In addition, BRCA1 depletion promoted the recruitment of DNMT1/3a/3b and the enrichment of the EZH2-mediated transcriptional repressive epigenetic marks H3K27me3 on the FOXO3 promoter showing crosstalk between the genetic and epigenetic changes in BC [53].

H3K27 methylation was also linked to metastasis as EZH2 mediates transcriptional silencing of EMT-suppressor genes: *CDH1* that encodes the E-cadherin and *RKIP* (Raf-1 kinase inhibitor protein) [54,55]. The expression of both *CDH1* and *RKIP* is directly repressed by the EMT inducer Snail TF in prostate and breast cancers [56,57]. A DNA motif called an E-box (enhancer box) that possesses the consensus hexanucleotide core sequence of CANNTG (where N is any nucleotide) at the promoters of the above genes is crucial for EZH2-mediated gene silencing. In EZH2-overexpressing breast epithelial cells, reduced CDH1 expression and elevated H3K27me3 levels were observed in the gene promoter. Interestingly, HDAC activity was shown to increase EZH2 enzymatic activity for H3K27me3 at the CDH1 promoter. The prior removal of acetyl groups from H3K27 by HDAC enabled EZH2-controlled histone methylation, causing a reduction in CDH1 expression, and leading to metastasis and invasion in BC [54]. Similarly, EZH2 negatively regulated RKIP transcription by increasing the level of histone marks associated with repression. Using a combination of loss and gain of function approaches, the direct recruitment of EZH2 and SUZ12 to the proximal E-boxes of the RKIP promoter with simultaneous enrichment of H3K27me3 and H3K9me3 was shown in metastatic and non-metastatic breast and prostate cancer cell lines. The repressing activity of EZH2 on RKIP expression was dependent on HDAC recruitment to the gene promoter and was negatively regulated upstream by microRNA, *miR-101* [55].

The H3K27me3 repressive chromatin mark was also involved in the suppression of cell surface-expressed major histocompatibility class II (MHC II) molecules- the mechanism frequently used by tumour cells to escape immune system recognition and elimination by T-cells [58]. A significant increase in H3K27me3 was detected at the promoter IV (pIV) of the class II transactivator (CIITA) of MHC II upon interferon (IFN)-γ stimulation in the estrogen-independent MDA-MB-435 cell line. Moreover, a simultaneous decrease in TF recruitment to CIITApIV as well as its significantly increased occupancy by EZH2 was observed. CIITA functions by recruiting to MHC II promoter components of the basal transcriptional machinery, histone acetyltransferases (HATs), HDACs, chromatin remodeling complexes, and kinases that phosphorylate RNA polymerase II. The presence of H3K27me3 suppresses CIITA expression, thus reducing the CIITA expression thereby reducing MHC II molecules on the cancer cells’ surface of cancer cells and allowing cancer cells to evade the immunity network. Among other inflammation-related genes regulated by H3K27me3 are: *GAGE2* (tumour G antigen 2A), *KRT17* (keratin 17), *TNF* tumour necrosis factor (multifunctional pro-inflammatory cytokine), and *CCL2* (C-C motif chemokine ligand 2) [59].

In addition, bioinformatic de novo motif analysis of BC subtype-specific regions has identified the most enriched TFs occupied by H3K27me3 marks [60]. In MDA-MB-436 (TNBC), the top five enriched genes were *NFATC2* (nuclear factor of activated T cells 2), *TCFAP2* (TF AP-2α), *MYB1* oncogene, *TCF3*, *ZFP161* (zinc finger protein 161). In SKBR3 (HER2+) cells: *PBX1* (PBX homeobox 1), *SOX8* (SRY-box TF), *ZFX* (zinc finger X-chromosomal), *HOXD13* (homeobox D13), *CHR* (cell cycle homology region) were identified. The *FOXH1*, *TCF3*, *IRF6* (interferon regulatory 6), *EOMES2* (T-box TF), *NHLH1* (helix loop helix 1) were found in ZR751 (luminal) cells. Furthermore, the high levels of H3K27me3 in HER2 overexpressed cell line at *ER* and *PR* reflected the repression of those two factors in HER2+ BC [60].

Based on those facts, H3K27 methylation occurs not randomly in the genome but rather is a targeted mechanism in which HMTs can be recruited to chromatin to modulate gene expression depending on the cellular context.

### 3.3. The Removal of H3K27me3 in BC

The enrichment of H3K27me3 might serve as a mark of the genes that need to be regulated by other proteins acting as histone methylation readers/erasers in cancer cells, for example, promoters containing both H3K4me3 and H3K27me3 histone marks coincided with strong binding sites of PRDM14, a zinc finger protein containing the PR domain homologous to the SET domain of KMTs [61]. PRDM14 maintains stemness in embryonic stem via epigenetic regulation. In addition, its increased level was reported in samples from various cancers’ patients’ samples. In particular, the PRDM14 protein was present in 38% of BC patients’ biopsies, and its expression was associated with a poor prognosis for BC patients regardless of cancer stage. Moreover, PRDM14 expression was inversely correlated with ER expression but did not associate with PR or HER2 expression. In cancer cells, PRDM14 conferred stem cell-like phenotypes by regulation of the expression of genes involved in cancer stemness, metastasis, and chemoresistance in vitro. Some epigenetic changes, such as reduction of the DNA methylation of the proto-oncogene and stemness gene promoters, as well as enhanced methylation of tumour suppressor genes in cancer cells, were mediated by PRDM14. In addition, silencing of *PRDM14* expression reduced tumour size and metastasis in vivo, while conditional loss of PRDM14 function improved survival of MMTV-Wnt-1 transgenic mice, a spontaneous model of murine BC. Those findings revealed the potential of PRDM14 as a target for cancer stem cell therapy [61].

Recently, the significance of PRDM14 in the removal of H3K27me3 repressive marks was reported in migrating mouse primordial germ cells (PGC) [62]. While PGCs undergo extensive epigenetic reprogramming as well as the remodeling of the inactive X chromosome to enable X chromosome reactivation (XCR) in females, it was shown that the global upregulation of the repressive H3K27me3 mark was PRDM14 expression-dependent and it progressed with the developmental stage at an equal rate in both male and female embryos. According to previous studies, PRDM14 could recruit PRC2 to PRDM14 targets through binding to its partner and co-repressor CBFA2T2 (core-binding factor runt domain subunit A2 translocated to 2) /MTGR1 [63,64,65]. Moreover, PRDM14 was required to remove H3K27me3 from the inactive X chromosome during PGC migration. The authors suggested that PRDM14 could thereby regulate X-chromosomal H3K27me3-depletion by downregulating *Xist* (X-inactive specific transcript) lncRNA, as H3K27me3 accumulation on the X chromosome was shown to be *Xist*-dependent and PRDM14 binds to regulatory regions in *Xist* intron 1 and upstream of the *Xist* activator Rnf12 (ring finger protein 12) [66,67,68].

## 4. The Potential of Targeting H3K27 Methylation in BC Treatment

Targeting the cancer epigenome with inhibitors of chromatin-modifying enzymes or small inhibitory molecules to release silenced genes from the repressed state might become a powerful approach for cancer research and drug development. To erase the H3K27me3 mark from the regions of gene promoters, several approaches were undertaken, such as direct or indirect inhibition of EZH2, introducing synthetic TFs recognizing H3K27me3, using natural anticancer compounds or a combination of known anticancer drugs. However, targeting H3K27 methylation in BC treatment might cause possible adverse events related to side effects that might occur during treatment. Especially, EZH2 inhibitors which, in addition to preventing H3K27 methylation, could also block the activity of EZH2 related to its other functions. Therefore, the application of the H3K27 methylation targeting agents need proper validation to address also non-methylation-related effects.

### 4.1. EZH2 Inhibitors

One of the known PRC2 inhibitors is 3-deazaneplanocin A (DZNep), which causes apoptotic cell death in cancer cells (including MCF7 and MB-468 BC cells) but does not affect normal cells (eg MCF-10A) [69]. DZNep inhibits activity of S-adenosyl-L-homocysteine (SAH) hydrolase, indirectly inhibiting EZH2 by increasing SAH, which acts as a SAM antagonist thus blocking histone methyltransferase activity [70]. Moreover, DZNep has minimal activity on genes silenced by DNA methylation [69,70]. In a variety of cancer cell lines (including MB-468 BC cells), treatment with DZNep effectively inhibited H3K27 methylation (but not H3K9 methylation) through depletion of cellular levels of PRC2 components: EZH2, SUZ12, and EED [69]. The authors have shown that genes selectively repressed by PRC2 in BC can be reactivated by treatment with DZNep. Interestingly, in the BRCA1-deficient mammary tumour cells exhibiting overexpression of EZH2, DZNep caused apoptosis more effectively than in BRCA1-proficient mammary tumour cells [71]. Despite its interest as a candidate drug in anticancer therapy, very few reports have investigated potential adverse effects of DZNep in vivo, yet not in BC models. The preclinical study investigating acute toxicity reported that DZNep causes nephrotoxicity in a dose-dependent manner in rats [72]. Furthermore, as shown in male mice, DZNep had no effect on behavior and induced side effects were limited to the testes only [73]. Thus, more in vivo experiments on the application of this small molecule as an epi-drug in BC are highly expected. Following the DZNep another group of potent and highly selective EZH2 inhibitors was developed that act competitively with SAM [74]. The two, GSK926 and GSK343, were shown to decrease the histone H3K27me3 level and inhibit EZH2 activity in breast (HCC1806 TNBC) and prostate cancer (LNCaP) cells, yet GSK343 exhibited some limitations due to the high clearance (referred to as the volume of plasma from which a substance is completely removed per unit of time) in rat pharmacokinetic studies.

The reduction of H3K27me3 was also observed upon glycogen synthase kinase 3 beta (GSK3β) activation in MDA-MB-468, MDA-MB-435S, and BT-549 cells [75]. At the molecular level, GSKβ interacts with EZH2 in the cytosol and phosphorylates EZH2 at Ser363 and Thr367 in vitro, and activation of GSK3β upregulates EZH2 Thr367 phosphorylation in vivo. EZH2 phosphorylation suppresses its histone methyltransferase activity without changing the EZH2 protein level. Consequently, mutation of Ser363 and Thr367 in EZH2 to unphosphorylable Ala was associated with a higher level of H3K27me3 and led to enhanced ability of cell migration and anchorage-independent growth. Moreover, inactivation of GSK3β in tumour tissues of BC patients was positively correlated with a higher level of H3K27 trimethylation and the low levels of both were associated with better survival. The GSK3β’s tumour suppressory role was reported also in other types of tumours, which makes the enhancing GSK3β activity to inhibit EZH2 promiscuous in anticancer therapy [76]. On the contrary, in patients with early BC, increased GSK3β activity was associated with a poor prognosis suggesting the application of GSK3β inhibitors especially for the treatment of several subtypes of TNBC [77]. These results clearly show the importance of the potential drug validation by in vivo studies using patient-derived xenograft and genetically engineered preclinical mouse models, as targeting such multifaceted kinase in highly diverse BC could result in various side effects.

Interestingly, a member of the poly (ADP-ribose) polymerase family (PARP), PARP1 has been shown to interact with and down-regulate EZH2 expression, thereby reducing levels of H3K27me3 in MDA-MB-231 cells [78]. PARP acts in DNA repair processes in which it catalyzes the attachment of ADP-ribose (PAR) units to itself and its target proteins (PARylation) recruiting the DNA repair machinery to the site of DNA damage [79]. For example, during DNA single strand break (SSB) repair, inhibition of PARP causes the replication fork to stall during DNA replication and induces double strand break (DSB), which is further repaired by the homologous recombination (HR) repair pathway, requiring, among others, the BRCA protein [80,81]. Upon DNA damage caused by oxidative stress or alkylation, PARP1 PARylates EZH2 and induces dissociation of the PRC2 complex, EZH2 downregulation, followed by reduction of EZH2-mediated H3K27me3 [77]. In contrast, inhibition of PARP by specific inhibitors (PARPi) attenuates alkylating DNA damage-induced EZH2 downregulation, thereby promoting EZH2-mediated gene silencing and cancer stem cell properties compared to PARPi-untreated cells. Therefore, in BRCA-deficient BC, combinational treatment using PARPi (olaparib) and EZH2i such as GSK343 was studied [78]. This approach was previously used successfully in the treatment of BRCA1-deficient ovarian cancer [82]. As reported in BC (SUM149, HCC38) and ovarian cancer (UWB1.289) cells that were not responding to PARPi alone the addition of EZH2i can improve the therapeutic effect of PARPi [78]. These findings have delivered a strong argument to test this combination in clinical trials for patients with BRCA-defective tumours. In fact, the phase II clinical study for patients with HR+/HER2- endocrine-resistant advanced BC treated with SHR2554 (EZH2i) and SHR3162 (PARPi) is now recruiting participants (ClinicalTrials.gov Identifier: NCT04355858).

### 4.2. H3K27me3 Binding TFs

EZH2 inhibitors indirectly target H3K23me3, but could also inhibit the activity of other EZH2 targets. This obstacle was overcome by the development of an inhibitor that directly targets H3K27me3 within the chromatin fiber, namely a polycomb-based transcription factor (PcTF), a fusion activator that targets H3K27me3 marks via its N-terminal motif and recruits endogenous transcription factors into PRC-silenced genes [83]. The effectivity of this approach was studied in BC model cell lines: MCF-7 (luminal A), BT-474 (luminal B), BT-549 (basal-like) with elevated levels of both EZH2 and H3K27me3. Overexpression of PcTF in BC cells upregulated dozens of genes, including a known set of 19 genes in the interferon response pathway, just 24 h after transfection. Interestingly, the highest degree of PcTF sensitivity was observed in BT-549 cells. Additionally, PcTF-sensitive genes associated with a bivalent chromatin environment and moderate levels of basal transcription and did not overlap with very strongly repressed, PRC-enriched loci, which might suggest that PcTF acts not specifically or that the occupation ofH3K27me3 reflects genes with very low expression, not in those completely silenced, as shown in murine cells [84].

### 4.3. DNMT and HDAC Inhibitors

On the other hand, in BC subtypes with the lowest H3K27me3 status correlated with a poor prognosis such as TNBC, an attempt to up-regulate the level of this chromatin mark to improve the outcome could be considered. As H3K27 methylation was shown to crosstalk with other chromatin modifications, a combinatorial treatment with DNMTi (guadecitabine/SGI-110) or HDACi (MS275) was tested in the developed TNBC model cell lines that exhibit highly tumorigenic and metastatic characteristics, XtMCF and LmMCF cells [85]. The application of these two drugs alone resulted in an elevated H3K27me3, while the combined treatment synergistically increased H3K27me3. Treatment caused transcriptional reprogramming manifested by the inhibition of EMT promoting protein-mutant p53 (frequently found in cancer cells), ZEB1 and EZH2, and induction of E-cadherin, apoptosis, as well as H3 trimethylation. The reversal of EMT resulted in cancer cell growth, colony formation, and stemness inhibition. Moreover, MS275 alone, and in combination with SGI inhibited xenograft growth of XtMCF cells, while MS275 reduced LmMCF cell lung metastases in mice [85]. Together, these data support the notion that epigenetic reprogramming of EMT, including H3K27 methylation, can suppress the aggressive phenotype of TNBC cells.

### 4.4. Natural Compounds

In addition to synthetic compounds, many natural agents have been reported to exhibit anticancer activity in vitro and in animal models [86]. Among such compounds that affect H3K27 methylation in BC cells is emodin, a trihydroxyanthraquinone, an ingredient of several Chinese herbs (e.g., Rhubarb, *Rheum palmatum*). While for the initiation and development of solid tumours, including BC, tumour cells recruit and educate macrophages towards the anti-inflammatory M2-like phenotype, emodin attenuated tumour growth by inhibiting macrophage infiltration and M2-like polarization, accompanied by increased T-cell activation and reduced angiogenesis in mice bearing EO771 or 4T1 breast tumours [87]. Emodin inhibited several signalling pathways (IRF4, STAT6, and C/EBPβ) and increased H3K27me3 at the promoters of M2-related genes in tumour-associated macrophages- arginase 1 (M2 marker) and TFs: C/EBPβ (CCAAT/enhancer-binding protein β) and IRF4 (interferon-regulatory factor) without changes in global levels of H3K27 methylation. In addition, emodin inhibited macrophages migration toward and adhesion to tumour cells [87]. These results suggest that emodin can inhibit BC growth and metastasis acting on macrophages through H3K27me3- mediated gene silencing and effectively blocking the tumour-promoting macrophage infiltration.

Additionally, soy phytoestrogens: genistein, daidzein, and equol (daidzein metabolite) have been reported to interact with epigenetic modifications, for example, hypermethylation of tumour suppressor genes in vitro [88]. In two BC cell lines, MCF-7 and MDA-MB 231, phytoestrogens induced a decrease in trimethylated marks (H3K27me3, H3K9me3, H3K4me3) and an increase in acetylation marks (H4K8ac and H3K4ac) studied at six selected genes known to be up-regulated (*EZH2*, *SRC3*, *P300*) or down-regulated in BC (*BRCA1*, *ERα*, *Erβ*), and therefore, its action seems not to be gene-specific and independent of the BC subtype. Yet, only equol treatment resulted in a different H3K27me3 status in MCF-7 (decreased) and MDA-MB 231 (not changed) in all genes studied, and genistein in two genes (*Erβ*, *SRC* decreased in MCF-7 and did not change in MDA-MB 231) [88]. Therefore, more detailed studies of this natural compounds and thorough interpretation of the considering the cell type and gene-specificity will be crucial to evaluate their potential for BC treatment.

## 5. H3K27 Methylation in Cross-Talk with Other Chromatin Modifications and lncRNAs

The mentioned above studies revealed combinatorial histone PTMs present in BC. It was also confirmed by MS-based proteomic techniques used to compare global abundances of H3 and H4 PTMs from commonly used cell lines, mostly cancerous lineages, which showed the enrichment of different PTMs containing H3K27 methylation, especially in BC cell lines, MCF7 and MDA-MB-231 [37]. In these two cell lines, H3K27me3K36me1 and H3K9me3K14un (unmodified) histone marks were relatively enriched and positively correlated with the EZH2 expression. In addition, significant upregulation of a number of HMTs such as KMT5B, KMT2D (MLL2), NSD3 (nuclear SET domain-containing protein 3), PRDM16, MECOM (MDS1 and EVI1 complex locus protein) and NSD1 was observed in MCF7, MDA-MB-231, and pancreatic carcinoma PANC1 cell lines. Interestingly, EZH2 and NSD1 exhibited the greatest increase in hypermethylated states of H3K27K36 including H3K27me2K36me2, H3K27me3K36me2, H3K27me2K36me1, and H3K27me3K36me1. The enriched histone methylation marks in both EZH2 and NSD1 potentially suggest a functional relationship between these two methyltransferases. Moreover, the knockdown of the EZH2 in a mouse mammary xenograft model significantly reduced tumorigenicity in these animals, indicating that the level of H3K27 methylation affects BC formation [37].

### 5.1. Dual Modification Marks

The presence of dual modification marks such as H3K4me3 associated with the activation of nearby gene expression and H3K27me3 enriched in inactive gene promoters of cancer suppressor genes is not unusual [89,90]. This phenomenon called bivalency enables precise switching transcription on and off and it commonly occurs in stem cells, especially embryonic stem cells in which genes with bivalent promoters are poised to become either activated or repressed as the cell becomes more committed [89]. As cancer stem cells participate in tumour growth, invasion, and response to therapy, it became possible that bivalency also refers to cancer cells and is involved in oncogenesis [91,92]. In fact, the studies aimed to identify genes with a human embryonic stem cell (hESC)-like pattern of promoter-associated bivalency, genes that had a greater enrichment of H3K27me3 at promoters compared to gene body were determined in MCF7, MDA-MB-231, and MCF10A cells [91]. The pattern of H3K27me3 enrichment was observed at the gene promoters in the MCF7 cell line, while a more equal distribution of H3K27me3 was detected between the promoter and the gene body in MCF10A and MDA-MB-231 cells. In general, a strong relationship was observed between MCF7 and H7-hESC for both H3K4me3 and H3K27me3 bivalency patterns, suggesting that MCF7 cells are able to commit to a more embryonic-like state, losing mammary epithelial cell regulation and acquire to BC stem cell-like properties. Also, a comparison of distinct histone modification patterns between HMEC and three BC cell lines representing luminal, HER2+, and basal subtypes showed that subtype-specific histone modifications were involved in different epigenetic regulation programs and signalling pathways in BC progression [60]. Specifically, hundreds of subtype-specific bivalent promoters carrying both H3K4me3 and H3K27me3 marks were identified. Genes associated with distinct histone marks also exhibited subtype-specific expression profiles in BC cell lines and primary tumours in patients. Subtype-specific classification of the patients, together with the survival data, has shown that H3K27me3 proximal gene classifiers were significantly correlated with relapse-free survival outcomes in all basal-like and some HER2+ patients [60].

Recently, the effect of ER expression in HER2+ BC subtypes in the context of bivalency was studied. The bioinformatic analysis of the public data on H3K4me3 and H3K27me3 bivalency marks in gene promoters in HER2+/ER+ (MB361 and UACC815) and HER2+/ER- (AU565 and SKBR3) cell lines revealed the differences in the bivalently marked genes and pathways identified [91]. Not surprisingly, several pathways related to cancer progression and metastasis as well as tumour suppression were found to have been regulated by bivalency for example mTOR, Wnt, Rap1, PI3K-Akt, cAMP, and calcium signalling yet with different preferences depending on the ER status. Especially, genes involved in the HER signalling pathway were enriched in the HER2+/ER- cell lines, but not in the HER2+/ER+ cell lines, underscoring the role of HER signalling in HER overexpressing/ER negative cells [91].

### 5.2. DNA Methylation

The relation between epigenetic silencing via H3K27me3, H3K9me2, and DNA methylation was studied in human breast (MCF7), prostate (PC3), and colon (SW48) cancer cells, showing that H3K27me3 occurs independently of the promoter DNA methylation [93]. The H3K27me3 mark was found at 5% of DNA methylated genes’ promoters. Interestingly, genes silenced in association with promoter DNA methylation showed a slightly elevated enrichment for H3K27me but no H3K27me3. Conversely, the latter analyzes of DNA methylation and H3K27me3 in human breast, prostate, and colon cancer cell lines revealed that nearly 25% of DNA methylated genes had the dual modification in cancer cells, twice more than in normal cells [47]. Among the genes under control of these dual modifications were known tumour-suppressor genes such as IGFBP7 (insulin-like growth factor binding protein 7) and SFRP1 (secreted frizzled-related protein 1), and callponin 3 (CNN3) associated with cytoskeleton maintenance. Combined treatment with a DNA demethylating agent, 5-aza-2′-deoxycytidine (5-aza-dC), and an EZH2 inhibitor, GSK126, induced marked re-expression of genes with the dual modification and showed an additive inhibitory effect on cancer cell growth in vitro, suggesting the possible therapeutic application of the inhibitors used [47].

### 5.3. LncRNAs

Not only histone PTMs and DNA methylation, but also ncRNAs were shown to affect chromatin structure. In particular, an emerging class of lncRNAs (possessing more than 200 nucleotides) with members are able to interact with both DNA and chromatin-modifying proteins at the same time, creating scaffolds for DNA-protein complexes acting in gene activation or repression [94,95]. The activity of lncRNAs was associated with many types of cancer including BC [96,97,98]. For example, a lncRNA *HOTAIR* (homeobox, HOX antisense intergenic RNA) was overexpressed in ER+ BC cells (MCF7) resistant to the estrogen antagonist drug tamoxifen, in which it promoted the proliferation, while *HOTAIR* depletion by shRNA lentiviral transduction considerably reduced cell survival and decreased growth during anti-hormone therapy with tamoxifen [99]. Mechanistically, *HOTAIR* recruits the PRC2 complex to specific loci across the genome, resulting in altered H3K27 methylation and epigenetic silencing of metastasis suppressor genes, thereby increasing cancer invasiveness and metastasis [99]. Another lncRNA, a metastasis-associated lung adenocarcinoma transcript 1 (*MALAT1*) that can silence gene expression through binding to the PRC2 complex followed by methylation of H3K27 in bladder cancer [100] was shown to be positively correlated with BC progression and metastasis in vitro and in vivo in the MMTV-PyMT mouse mammary tumor model of human luminal B BC [101]. Consistently, an elevated level of *MALAT1* in BC was correlated with an increased tumour size and stage, as well as poor prognosis for patients [100,101,102]. In contrast, a metastasis suppressive role of *MALAT1* in BC was recently reported in *Malat1* knockout mice and MDA-MB-231 cells [103]. Through comprehensive targeted inactivation, rescue and overexpression approaches in multiple in vivo models, the authors proved that *MALAT1* plays the antimetastatic role by inhibiting the TEAD (TEA domain family member) TFs, which together with their coactivators YAP (Yes-associated protein 1) and TAZ (TEAD 4) promote tumour progression and metastasis through the transcriptional activity [103]. Although these findings reveal new insights into the complex role of *MALAT1* in BC, their careful interpretation in relation to the cancer cell context is needed.

LncRNAs can also alter H3K27me3 status through modulating the stability of EZH2 as shown for *ANCR* alias *DANCR* (antidifferentiation/differentiation antagonizing ncRNA) [104]. *ANCR* interacts with EZH2, thus promoting the binding of CDK1 to EZH2 and phosphorylation at Thr-345 and Thr-487 residues of EZH2, resulting in the ubiquitination and subsequent degradation of EZH2. Repression of *ANCR* results in an increased H3K27me3 global level, especially at the promoter regions of EZH2 target gene (*CDH1*, *HOXA10,* and *DAB2IP* encoding Ras GTPase-activating protein), whereas ectopic *ANCR* expression decreases the H3K27me3 global level. *ANCR* inhibits BC cell migration and invasion in vitro in MDA-MB-231 and MDA-MB-453 (highly invasive and metastasizing ER cells) and in vivo in the mouse xenograft model. Similar data on the *ANCR-* mediated repression of tumorigenesis have recently been reported [105]. Yet, the authors showed that the *ANCR*-EZH2 interaction promotes the EZH2 binding to the promoter of suppressor of cytokine signalling 3 (*SOCS3*), increasing the level of H3K27me3 and inhibiting *SOCS3* expression, which is associated with enhanced BC cell migration and invasion in vitro and in vivo [105].

## 6. Discussion

For a long time, breast cancer was considered a disease with genetic origins, predominantly resulting from mutations in key genes such as *BRCA1* and *BRCA2*. Nonetheless, the mounting evidence from the last two decades shows that epigenetic changes are deeply involved in the progression of BC. The accumulation of epigenetic changes driven by DNA methylation, histone modifications, and the activity of lncRNAs in tumor-related genes may contribute to serious cancer outcomes, as genome-wide studies have shown that chromatin-mediated epigenetic silencing in a great number of genes is strongly linked to a poor prognosis [44,58]. PcG- mediated gene silencing through H3K27 methylation is one of the most frequently observed events in cancer cells, especially at promoters of tumour suppressor genes as well as genes related to inflammatory and immunological response. Yet, it is not completely clear how this chromatin mark functions in gene repression and how it is controlled. Global gene methylation level analyzes in BC have shown the association of the enriched H3K27me3 signature with luminal A, while in luminal B, TNBC, and ER-positive tumours with high proliferation, low levels of this mark were reported [37,38,39,40]. Additionally, H3K27 me3 loss was linked with a poor prognosis for BC patients [43]. The low abundance of H3K27me3 was proposed to be simply indicative of there being relatively fewer targets of polycomb gene targets in basal-like cancer subtypes compared to the number of genes under PcG control in more differentiated breast cancer subtypes [40]. Furthermore, the low abundance of H3K27me3 might be explained by the fact that the PcG targets in both undifferentiated and differentiated cells are silenced instead by DNA methylation as shown in luminal B tumours [40]. However, this phenomenon still needs more comprehensive investigation.

Seeing the bigger picture, H3K27me3 is often accompanied by other chromatin marks, for instance, H3K4ac in BC. The bivalency observed in the gene promoter regions allows for precise tuning of gene transcription and reflects the plasticity of the cancer (epi)genome. The involvement of lncRNAs recruiting PcG proteins and/or TFs makes the regulation of the cancer genes even more complex and as lncRNAs are currently intensively explored, it seems a matter of time when new mechanistic data on their crosstalk with H3K27 methylation in gene regulation will appear. 

One of the intriguing issues is also the co-existence of three different H3K27 methylation states. Although introduced by the same enzyme (EZH2) (with the additional impact of G9a on H3K27 monomethylation), the functions of H3K27me3, H3K27me2, and H3K27me1 appear to be different. As mentioned above, H3K27me3 correlates with gene silencing, but the function of H3K27me2 function seems not to be related to transcription at least in human hematopoietic stem cells (probably due to its abundance and widespread distribution throughout the genome), and H3K27me1 is more associated with transcribed regions and enhancers [106,107]. Therefore, understanding this diversity in BC is of great value and will advance our ability to predict BC patients’ outcomes and effectively treat BC in the future as targeting epigenetic modifications in cancer cells is a promiscuous strategy. Several agents acting by removing the H3K27me3 mark were developed, mostly EZH2 inhibitors exhibiting anticancer activity. Their effectiveness and safety were tested in clinical trials for the treatment of various types of cancer (for review see [108]), yet its evaluation in BC patients is still missing. Furthermore, the direct targeting of H3K27me3 in BC remains poorly explored. Lastly, since epigenetic regulation during tumorigenesis is complex, a better outcome of combinatory treatment using H3K27me3- targeting agents with inhibitors of other chromatin marks is expected.

## 7. Conclusions

In conclusion, H3K27me3 functions as a repressive chromatin mark, which can be used as a biomarker for BC prediction of the outcome of BC as high levels of H3K27me3 associated with luminal A BC subtype and lower tumour grade, while low levels of H3K27me3 associated with luminal B subtype, large tumour size, and the presence of metastases in lymph nodes. Although several classes of agents that block H3K27 trimethylation have been developed, there is still substantial work needed to directly target this H3 PTM as a strategy for BC treatment.

## Figures and Tables

**Table 1:** Status of global H3K27 methylation marks studied in breast cancer subtypes. HR- hormone receptors, HER2- human epidermal growth factor receptor 2, Ki67- proliferation marker, ER- estrogen receptor, EZH2- enhancer of zeste homolog 2, ↑ upregulated, ↓ downregulated).

BC Subtype	Model	Cell Line/Tissue	Markers	H3K27me3 Status	Correlation	References
Luminal A	In vitro	MCF7	HR+/HER2-/Ki67 low	↑	K27me2 ↑	[37]
		T47D	HR+ HER2-			
	In vivo	Patients tumour tissues	ER+/HER2−/Ki67 low	↑	positive correlation with lower tumour grade; associated with high K27me3/K36me1, and K27me2/K36me1 and low levels of K9me3, K9me3/K14ac, K36me1, K36me2, and K27me1	[39,40,42]
Luminal B	In vivo	Patients tumour tissues	ER+/HER2−/Ki67 high	↓	positively correlated with EZH2 expression	[40]
TNBC	In vitro	MDA-MB-231; MDA-MB-453	HR-/HER2-	↑	K27me2 ↑	[37]
	In vivo	Patients tumour tissues	HR-/HER2-	↓	positively correlated with EZH2 expression; associated with higher levels of K27me1/K36me1 and K27me2/K36me2	[39,42]
HER overexpressed	In vivo	Patients tumour tissue	HER2+	↓	positively correlated with EZH2 expression; associated with higher levels of K27me1/K36me1 and K27me2/K36me2	[42]

## Data Availability

Not applicable.
